# MyD88 Is Not Required for Muscle Injury-Induced Endochondral Heterotopic Ossification in a Mouse Model of Fibrodysplasia Ossificans Progressiva

**DOI:** 10.3390/biomedicines9060630

**Published:** 2021-06-01

**Authors:** Huili Lyu, Cody M. Elkins, Jessica L. Pierce, C. Henrique Serezani, Daniel S. Perrien

**Affiliations:** 1 Division of Endocrinology, Metabolism, and Lipids, Department of Medicine, Emory University, Atlanta, GA 30322, USA; huili.lyu@emory.edu (H.L.); cody.elkins@emory.edu (C.M.E.); jessica.liane.pierce@emory.edu (J.L.P.); 2 Department of Endocrinology, Endocrinology Research Center, Xiangya Hospital of Central South University, Changsha 410000, China; 3 Division of Infectious Diseases, Department of Medicine, Vanderbilt University Medical Center, Nashville, TN 37232, USA; h.serezan@vumc.org

**Keywords:** IL-1β, muscle injury and repair, chondrogenesis, osteogenesis, *Acvr1*, fibroadipoprogenitor, FAP, MyD88

## Abstract

Excess inflammation and canonical BMP receptor (BMPR) signaling are coinciding hallmarks of the early stages of injury-induced endochondral heterotopic ossification (EHO), especially in the rare genetic disease fibrodysplasia ossificans progressiva (FOP). Multiple inflammatory signaling pathways can synergistically enhance BMP-induced Smad1/5/8 activity in multiple cell types, suggesting the importance of pathway crosstalk in EHO and FOP. Toll-like receptors (TLRs) and IL-1 receptors mediate many of the earliest injury-induced inflammatory signals largely via MyD88-dependent pathways. Thus, the hypothesis that MyD88-dependent signaling is required for EHO was tested in vitro and in vivo using global or Pdgfrα-conditional deletion of MyD88 in FOP mice. As expected, IL-1β or LPS synergistically increased Activin A (ActA)-induced phosphorylation of Smad 1/5 in fibroadipoprogenitors (FAPs) expressing Alk2^R206H^. However, conditional deletion of MyD88 in Pdgfrα-positive cells of FOP mice did not significantly alter the amount of muscle injury-induced EHO. Even more surprisingly, injury-induced EHO was not significantly affected by global deletion of MyD88. These studies demonstrate that MyD88-dependent signaling is dispensable for injury-induced EHO in FOP mice.

## 1. Introduction

Fibrodysplasia ossificans progressiva (FOP) is a rare congenital disease caused by one of several point mutations in the BMP type I receptor, ALK2 [1,2] and results in episodes of hyperinflammatory, fibroproliferative, and edematous soft tissue lesions, termed “flares”, that frequently progress to intramuscular endochondral heterotopic ossification (EHO) [3,4,5,6]. Heterotopic ossification (HO) is a pathological process of extraskeletal osteogenesis in muscle and soft tissue. Over time, HO progressively replaces skeletal muscles, typically leading to joint ankylosis, disfigurement, pain, and loss of mobility [3,5,7,8]. In addition to FOP, EHO is also a common clinical complication of musculoskeletal trauma and other rare genetic diseases. Unfortunately, there are currently no approved effective treatments for FOP, and knowledge of the underlying mechanisms is very limited.

The most common FOP-causing mutation is *ACVR1*^617G>A^, which encodes ALK2^R206H^ [1]. The expression of Alk2^R206H^ in mesenchymal-like fibroadiprogenitors (FAPs) residing in skeletal muscle is necessary and sufficient for injury-induced EHO in mice [9,10]. While other potential progenitor cell linages have been described for both FOP and acquired heterotopic ossification [11,12,13,14,15,16,17,18,19], FAPs appear to be the primary chondrocyte progenitor in FOP lesions [9,10,20]. The ALK2^R206H^ mutation causes hypersensitivity to its cognate osteogenic BMP ligands and, more importantly, confers neofunctional sensitivity to aberrant Activin A (ActA)-induced signaling [21,22]. A member of the TGFβ superfamily, ActA is the requisite ligand for EHO formation in FOP mice [10,21,22,23], and an anti-activin A antibody, garetosumab, is currently in clinical trials for the treatment of FOP. Despite these key discoveries, it is clear that many other cell types and signaling pathways contribute to the complex process of EHO in FOP.

HO has been described by some as a form of failed or misdirected tissue repair. Indeed, whether caused by musculoskeletal trauma, burns, or FOP, EHO almost uniformly begins with inflammation, and the depletion of mast cells or macrophages reduces intramuscular EHO in multiple mouse models [24,25,26], including Alk2^R206H^-expressing FOP mice [25]. Consequently, the roles of inflammatory cytokines and related signaling pathways are of great interest in the field.

Although the underlying cause of FOP is inappropriate activation of the Smad1/5/8 pathway by ActA/ALK2^R206H^ signaling, the kinetics and transcriptional effects of this aberrant signal may still be modulated by crosstalk with other pathways. Indeed, inflammatory cytokines and signaling pathways, including toll-like receptors (TLRs) and their ligands, can enhance BMP-induced Smad1/5/8 signaling in Alk2^wt^ cells [27,28,29,30] and cells from FOP patients [31].

Toll-like receptors (TLRs) are a group of widely expressed transmembrane receptors activated by “debris” of damaged and necrotic cells and/or pathogens (pathogen-associated molecular patterns, PAMPs). The release of damage-associated molecular patterns (DAMPs) is one of the first consequences of cell and tissue damage. DAMPs, including CpG DNA fragments, S100A1, and ribosomal RNA, activate TLRs on neighboring cells and distant innate immune cells, leading to inflammatory immune cell activation and migration. DAMP-induced TLR signals are among the first inflammatory signals in damaged tissues, and TLR signaling has been shown to regulate ActA expression [32,33,34,35] and enhance canonical BMP signaling [31,36,37].

One study using patient-derived FOP fibroblasts reported that TNFα increases the expression of TLRs 3–10, and TNFα-induced expression of TLRs 1, 2, 3, 4, and 6 is significantly greater in FOP fibroblasts than in WT fibroblasts [31]. They also found the classical TLR4 agonist, LPS, and TLR3 agonist, polyIC, enhance BMP signaling at the level of type 1 receptor activity via the TLR signaling intermediate ECSIT [31], which is a downstream component of MyD88-dependent pathways activated by both TLRs and IL1 receptor 1 (IL1R1). The results of these studies suggest the adapter protein MyD88, which is required for most TLR- and IL1R1-induced signaling, may be important for interactions between TLR and canonical BMP signaling. However, crosstalk of these pathways has not been reported in the context of aberrant ActA/Alk2^R206H^-induced signaling. Hence, the studies reported here were designed to test the hypothesis that MyD88-dependent signaling enhances ActA-induced signaling of Alk2^R206H^ and is required for muscle injury-induced EHO in FOP mice. These studies utilized tamoxifen-induced ubiquitous or conditional deletion of MyD88 in *Alk2^R206H^*-expressing mouse models of FOP to demonstrate that MyD88 is unexpectedly dispensable in the growth, maturation, and remodeling of EHO lesions.

## 2. Materials and Methods

### 2.1. Animal Husbandry and Breeding

All studies were approved by the Institutional Animal Care and Use Committee at Emory University (protocol 201900179, approved 4 November 2019) or Vanderbilt University Medical Center (protocol M1800121, Approved 8 October 2018) and followed ARRIVE guidelines. Mice were housed in standard cages with free access to food and water and a 13 h on/11 h off light schedule. *Acvr1^R206H_FlEx^* (VG1649) mice [22] were generously provided by Regeneron Pharmaceuticals, Inc. (Tarrytown, NY, USA) through a research agreement. *Acvr1^R206H_mg^* mice were designed by this lab, created at Ozgene, Inc. (Perth, Australia), and are described in detail in another pending publication [38]. *Myd88-floxed* (*Myd88^f/^*^f^) (*B6.129P2(SJL)-Myd88^tm1Defr^/J*, Stock Number 008888), *R26^cE2^* (*B6.129-Gt(ROSA)26Sor^tm1(cre/ERT2)Tyj^/J*, Stock Number 008463), and *Pdgfra^cE2^ (B6.129S-Pdgfra^tm1.1(cre/ERT2)Blh^/J*, Stock Number 032770) mice were purchased from The Jackson Laboratory (Bar Harbor, ME, USA) and crossed with *Acvr1^R206H_FlEx/+^* or *Acvr1^R206H_mg^* mice to create the following lines: *R26^cE2/cE2^;Acvr1^R206H_FlEx/w^;Myd88^w/w^* and *R26^cE2/cE2^;Acvr1^R206H_FlEx/+^;Myd88^f/f^* (respectively dubbed *Acvr1^R206H_FlEx^;Myd88^w/w^* and *Acvr1^R206H_FlEx^;Myd88^−/−^* after tamoxifen-induced recombination), *Pdgfra^cE2/w^;Acvr1^R206H_FlEx/w^;Myd88^w/w^* and *Pdgfra^cE2/w^;Acvr1^R206H_FlEx/w^;Myd88^f/f^* (respectively dubbed *Acvr1^R206H_FlEx^;Myd88^w/w^* and *Acvr1^R206H_FlEx^;Myd88_Pdgfra−/−_* after tamoxifen-induced recombination), *or Pdgfra^cE2/w^;Acvr1^R206H_mg/R206H_mg^;Myd88^w/w^* and *Pdgfra^cE2/w^;Acvr1^R206H_mg/R206H_mg^;Myd88^f/f^ (Acvr1^R206H_mg^;Myd88^w/w^* and *Acvr1^R206H_mg^;Myd88_Pdgfra-/-_* after tamoxifen-induced recombination). Primers used for genotyping are listed in Table S1. Administration of tamoxifen (tam) to *R26^cE2/cE2^* mice results in “global” recombination of either of the *Acvr1^R206H^* conditional alleles and deletion of *Myd88^fl^* alleles, while *Pdgfra^cE2^* limits tam-induced recombination to mesenchymal-like stem cells, which include only FAPs and pericytes in skeletal muscle [10,20,39].

### 2.2. Tamoxifen Treatment and Induction of Heterotopic Ossification in FOP Mice by Pinch Injury

Mice were weaned at 21 days old and given ad libitum access to standard chow before being switched to tamoxifen-doped chow (TD.130856, Envigo, Indianapolis, IN, USA), formulated to deliver ~40 mg/kg/d for 8 days starting at 24–36 days of age. Tamoxifen chow was replaced with standard chow for 2 days before the mice were injured.

To induce EHO, all mice received bilateral pinch injuries of their lower hind limb muscles at 34–46 days of age. Mice were deeply anesthetized with 2–4% isoflurane in pure O_2_ and were monitored throughout the procedure. Each mouse received 0.5 mg/kg buprenorphine SR (ZooPharm, Rx 221356, Lot# BSRLAB0.5-201103, Fort Collins, CO, USA) prior to muscle injury. The lower hindlimb muscles were injured as previously described by this and other labs [40]. In brief, the mice were laid prone or supine, depending on the leg to be injured, and the midsection of the gastrocnemius and adjacent muscles was localized using forceps by gently squeezing the muscle group from the posterior side of the tibia. Once localized, the forceps were squeezed until there was a 1–2 mm gap between its arms, and pressure was maintained for 5 s. Care was taken to prevent movement or rotation of the forceps during the procedure, so as to prevent fracturing the fibula.

### 2.3. Radiographs and Microcomputed Tomography (microCT)

HO formation was monitored by in vivo plain radiographs acquired weekly after crush injury. Radiographs were acquired using an XPERT 40 digital radiography cabinet (Kubtec, Stratford CT, USA) at 40 kv and 1000 µA. The HO area in each leg was semiquantified using ImageJ version 1.52p. All mice were humanely euthanized 21 days post-injury (dpi) by exsanguination and cervical dislocation under deep isoflurane anesthesia. Hind limbs were excised, the skin carefully removed, and fixed in 10% neutral buffered formalin for 48 h before transfer to 70% EtOH.

Ex vivo microCT scans of each leg were acquired using a μCT40 or μCT50 (Scanco Medical AG, Wangen-Brüttisellen, Switzerland). All samples within each study were scanned on only one of the instruments. The μCT40 images were acquired at 70 kV, 114 μA, and a 16 mm FOV, with 500 projections/rotation, an integration time of 300 ms, and reconstructed with an isotropic voxel size of 16 μm. Images from the μCT50 were acquired at 55 kV, 200 μA, and a 14 mm FOV, with 500 projections/rotation, 250 ms integration time, and reconstructed with 13.3 μm isotropic voxels. IPL v5.42 (Scanco Medical AG, Wangen-Brüttisellen, Switzerland) was used to employ a semiautomated algorithm adapted from Buie et al. [41] to create a tight outer contour of the EHO lesions that defined the total lesion volume (including mineralized bone and the unmineralized interior of the HO), and the mineralized EHO volume was determined using a standardized density threshold and noise filter. A cartoon of the workflow is provided in Figure S1. In brief, hand-drawn contours were used to create a boundary around the HO and precisely separate the HO from the host leg bones. The manual contour was used as an outer boundary in the subsequent automated creation of a contour that tightly outlined the perimeter of the mineralized ossicles while excluding the tibia and fibula. The total volume of the resulting mask is deemed “lesion volume” (LV) and includes both mineralized and unmineralized tissues in the ossicles. The mineralized portion of the HO within the lesion mask, referred to as mineralized volume (MV), was segmented using a threshold of 250 mg HA/ccm, sigma 0.3, and support 2.

### 2.4. Histology

After microCT scanning, the hind limbs were decalcified in 20% EDTA on a shaker at room temperature for 14 days. Samples were dehydrated through graded ethanol, cleared in xylene or methyl salicylate, infiltrated, and embedded in paraffin. A standard rotary microtome (RM2255, Leica Biosystems, Wetzlar, Germany) was used to cut 5 μm transverse sections that were stained with hematoxylin-eosin and examined under brightfield microscopy to evaluate muscle repair and the composition of EHO.

### 2.5. Isolation and Culture of Intramuscular Fibroadipoprogenitors

#### 2.5.1. Skeletal Muscle Digestion

To isolate skeletal muscle FAPs, 6 weeks old male mice were sacrificed under deep isoflurane anesthesia. Skeletal muscles were harvested from hind limbs and forelimbs. Tendons and ligaments were thoroughly removed from the muscle, and the cleaned muscle was weighed. Muscle tissue was divided into 0.5 g portions that were placed into individual 50 mL conical tubes containing 2.35 mL of “harvest media” (DMEM w/o phenol Rred [+] 4.5g/L glucose, [+] L-glutamine, sodium pyruvate, and 2% pen-strep) at 37 °C. Samples were moved to a sterile biosafety cabinet, where they were finely diced using sterile scissors. An enzyme cocktail of 100 μL Enzyme D, 50 μL Enzyme R, and 12.5 μL Enzyme A from the Miltenyi multitissue dissociation kit (Order no. 130110201, Miltenyi Biotec, Bergisch Gladbach, Germany) was added for every 0.5 g of muscle. The muscle was digested inside in shaking incubator at 200 rpm set to 37 °C for 1 h and then triturated using 25 and 10 mL serological pipets. The muscle was returned to the shaker for 30 min and triturated again until the suspension smoothly passed through the tip of a 10 mL serological pipet. The muscle cell suspension was filtered through 70 and 40 μm cell strainers (cat. num. 22,363,548 and 22363547, Fisher Scientific, Waltham, MA, USA), and the flow-through was centrifuged at 400 rcf for 20 min at 4 °C.

#### 2.5.2. MACS and FACS

Cell pellets were incubated on ice for 15 min with 80 μL of ice-cold autoMACS rinsing solution (Order no. 130091222, lot:5180904365, Miltenyi Biotec, Bergisch Gladbach, Germany) and 20 μL of depletion cocktail (Adipose progenitor isolation kit Order no. 130106639, lot:5190809170, Miltenyi Biotec), diluted to 2 mL with additional cold MACS buffer, and passed through a Miltenyi MACS LD column (Order no. 130042901, Lot:5190524007, Miltenyi Biotec). The flow-through containing CD45-/CD31-/TERT- cell fraction, containing FAPs, was collected into sterile conical tubes for further staining and FACS. The CD45+/C31+/TERT+ fraction of the cell suspensions was flushed from the columns and used as unstained FACS control cells after red cell lysis with ACK buffer for 1.5–2 min at room temperature.

Suspensions of CD45-/CD31-/TERT- cells from mice of the same genotype were pooled and centrifuged at 400 rcf for 5 min at 4 °C. Detailed antibody information is in Table S2. Cell pellets were resuspended in 200 μL of MACs buffer containing 10 μL of PE-labeled anti-PDGFRa Miltenyi REA antibody (Order no. 130-122-019, Miltenyi Biotec) and 6 μL of APC-labeled anti-Sca1 Miltenyi REA antibody (Order no. 130-123-848, Miltenyi Biotec) per gram of initial muscle tissue and incubated in the dark for 30 min. Cells were washed with 3 mL of FAP harvest media, pelleted, and finally resuspended in 300 μL of FAP harvest media for flow sorting. PDGFRa and Sca-1 positive cells were sorted into 15 mL conical tubes containing 5 mL of FAP growth media (DMEM containing 4.5 g/L glucose, L-glutamine and sodium pyruvate, 10% FBS, 1% P/S, and 10 ng/mL FGF2 (R&D Systems, Minneapolis, MN, USA) by FACS Aria II (BD Biosciences, San Jose, CA, USA). The FACS gating strategy is shown in Figure S2.

#### 2.5.3. FAP Plating and Culture during Expansion

FAPs from *R26^cE2/cE2^;Acvr1^w/w^* or *R26^cE2/cE2^;Acvr1^R206H_FlEx/+^* mice were plated in 1 well of a 24-well standard tissue culture plate (Corning, Tewksbury, MA, USA) in FAP growth media, and the media was changed every 3 days until the cells reached 70–80% confluence. The cells were then trypsinized and replated at 6000–10,000 cells/cm^2^. After 3–4 passages, the cells were treated with 500 nM 4-OH tamoxifen (SLBZ7608, Sigma-Aldrich, St. Louis, MO, USA) for 48 h to induce cre-mediated recombination (Figure S2). Following 24 h of recovery from 4-OHT treatment, the cells were passaged and replated at 6000 cells/cm^2^ in 6-well plates and grown to 70–80% confluence for experimental use.

### 2.6. FAP Treatments and Measurement of Smad1/5 Phosphorylation by Western Blot

FAPs were serum starved in DMEM with 0.1% FBS, without FGF2 for 14–18 h. Then, experimental treatments were added according to the durations indicated in the corresponding figures. Cells were lysed using RIPA lysis buffer (R0278, Sigma-Aldrich, St. Louis, MO, USA) containing 1% protease inhibitor cocktail (P8340, Sigma), 5% protease inhibitor cocktail (P8465, Sigma-Aldrich), 1% phosphatase inhibitor Cocktail 2 (P5726, Sigma-Aldrich), and 1% phosphatase inhibitor cocktail 3 (P0044, Sigma-Aldrich). The samples were centrifuged at 10,000 rcf for 15 min at 4 °C, and the supernatant was collected and stored at –20 °C. Protein concentration was measured by BCA Protein Assay Kit (23225, Thermo Fisher Scientific, Waltham, MA, USA). In total, 8.4 ng of protein per sample was separated by PAGE in 10% acrylamide gels (4561033, Bio-Rad Laboratories, Hercules, CA, USA) and transferred to PVDF membranes using Trans-Blot Turbo Transfer System (Bio-Rad). PVDF membranes were blocked with 5% BSA in TBST (0.1% Tween 20) for 1 h at room temperature. The membranes were then incubated with primary antibodies for p-Smad1/5, Smad1/5, or β-actin overnight at 4 °C, followed by incubation with appropriate HRP-linked secondary antibodies for 1 h at room temperature. Detailed antibody information is in Table S3. Labeled protein was detected by chemiluminescence (Western Lightning Plus-ECL, PerkinElmer, Waltham, MA, USA) using the ChemiDocMP imaging system (Bio-Rad), and bands were quantified using ImageLab version 6.1 (Bio-Rad).

### 2.7. Statistical Analysis

Data sets that passed tests of normality and equal variance were analyzed by Student’s *t*-test, one-way, or two-way ANOVA with Tukey’s post hoc test and are presented as mean ± SEM. Data that did not display normal distribution or equal variance were analyzed by the corresponding nonparametric test and post hoc test and are presented with median and interquartile ranges. The specific method used for each data set is described in the respective figure legend. A *p*-value < 0.05 was considered statistically significant.

## 3. Results

### 3.1. Activation of MyD88-Dependent Signaling Pathways Enhances Aberrant ActA-Induced Smad1/5 Phosphorylation in FOP FAPs

**Figure 1:**
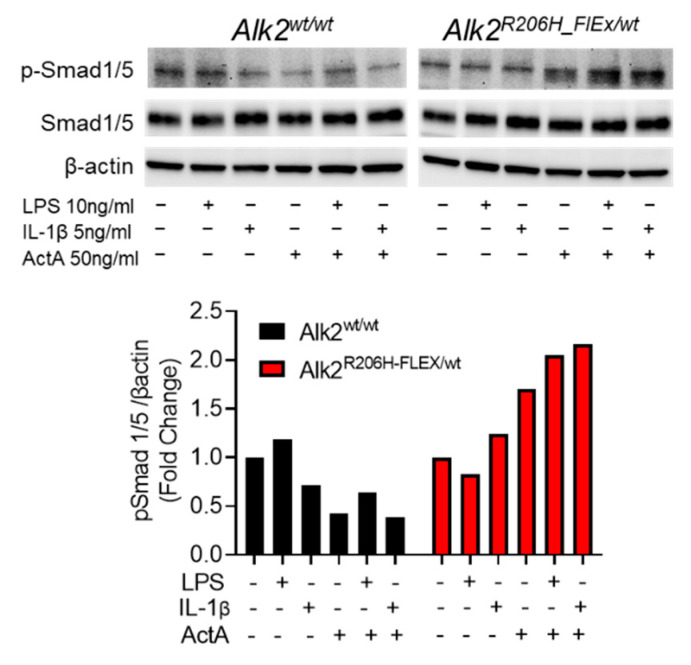
ActA-stimulated canonical BMP signaling in FAPs from FOP mice is enhanced by TLR or IL1R1 ligands. FAPs harvested from the hindlimb muscles of mice with the indicated genotypes were expanded in the presence of 10 ng/mL rhFGF2, serum starved overnight, then stimulated with the indicated ligands for 15 min. In *Alk2^wt/wt^* FAPs, pSmad1/5 was unaffected by LPS or ActA alone but decreased by IL-1β alone or in combination with ActA. However, in FAPs expressing *Alk2^R206H^*, pSmad1/5 was increased by ActA alone, and this was further increased by addition of LPS or IL-1β.

To determine whether TLR or IL1R1 signaling enhances ActA/Alk2^R206H^ signaling, phosphorylation of Smad1/5 was measured in murine *Alk2^w/w^* and *Alk2^R206H_FlEx/w^* FAPs treated with ActA alone or in combination with LPS or IL-1β (Figure 1). As expected, treatment of serum starved FAPs with LPS or IL-1β did not alter pSmad1/5, regardless of Alk2^R206H^ expression. In *Alk2^w/w^* FAPs, ActA decreased endogenous pSmad1/5, regardless of costimulation with LPS or IL-1β. However, in *Alk2^R206H/w^* FAPs, ActA increased pSmad1/5, which was further enhanced by cotreatment with LPS or IL-1β (Figure 1). This confirms that ligands which activate MyD88-dependent signaling pathways can enhance aberrant ActA/Alk2^R206H^-induced pSmad1/5 in a manner similar to that reported for BMP-induced signaling in *Alk2^wt^* cells [27,28,29,30] and cells from FOP patients [31].

### 3.2. Conditional Deletion of MyD88 in Pdfgrα-Positive Cells Does Not Alter the Volume of Injury-Induced EHO

Although previously published studies and the data in Figure 1 consistently demonstrate that activation of MyD88-dependent signaling enhances canonical signaling of type 1 BMP receptors in vitro, a role for these interactions in EHO has not been reported. Activation of MyD88-dependent signaling has complex, pleiotropic effects on numerous cellular processes including activation, differentiation, and apoptosis that are largely dependent on cell type [42,43,44,45,46,47]. Intramuscular FAPs are the primary source of chondrogenic progenitors in FOP mice [9,10] and have previously been targeted in FOP mice using *Pdgfra^cre^* [10,20,48]. Thus, *Pdgfra^cE2^;Acvr1^R206H_FlEx^;Myd88^w/w^* and *Pdgfra^cE2^; Acvr1^R206H_FlEx^;Myd88^f/f^* mice (respectively, *Acvr1^R206H_FlEx^;Myd88^w/w^* and *Acvr1^R206H_FlEx^;Myd88_Pdgfra−/−_*) were used to examine the role of MyD88-dependent signaling in FAPs during injury-induced HO formation.

Conditional recombination of both *Acvr1^R206H_FlEx^* and *Myd88-flox* alleles in intramuscular FAPs was confirmed in two ways: (1) using conventional PCR of gDNA from ear punches collected from mice before and after tamoxifen treatment and (2) using FAPs isolated from the skeletal muscle of mice after tamoxifen treatment. As expected, little or no recombination was detected in ear punches, while a high rate of in vivo recombination of both floxed genes was found in FAPs isolated from tamoxifen treated *Acvr1^R206H_FlEx^;Myd88_Pdgfra−/−_* mice (Figure S3A–C). Mice of both genotypes remained healthy and gained weight throughout 21 days post-injury (dpi); however, weight gain began to plateau during the third week due to HO-related restricted ambulation, which reduced access to food. The change in bodyweight was identical between genotypes except for a nonsignificant divergence at 21 dpi (Figure S3D).

**Figure 2:**
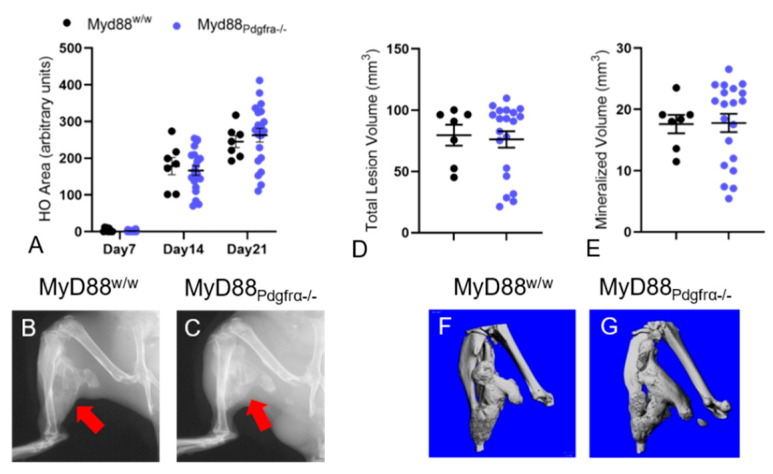
Conditional deletion of MyD88 in PDGFRα+ cells in *Acvr1^R206H_FlEx/wt^* mice did not affect muscle injury-induced EHO area and volume. (**A**) Semiquantification of intramuscular HO area in radiographs and (**D**,**E**) μCT-based quantification of HO volume demonstrated unaffected HO area (**A**), total lesion volume (**D**), and mineralized volume (**E**) in *Acvr1^R206H_FlEx^;MyD88_Pdgfrα−/−_* vs. *Acvr1^R206H_FlEx^;MyD88^w/w^* mice. Representative radiographs (**B**,**C**) and μCT reconstructions (**F**,**G**) of representative samples at 21 dpi illustrating extensive EHO (red arrows) in both genotypes. Statistical significance was tested using Kruskal–Wallis test or a two-tailed unpaired Student’s *t*-test. No significant differences were found.

Analysis of in vivo radiographs showed that the area of mineralized HO in *Acvr1^R206H_FlEx^;Myd88^w/w^* and *Acvr1^R206H_FlEx^;Myd88_Pdgfra−/−_* mice was not significantly different at both 14 and 21 dpi (Figure 2A–C). This was consistent with ex vivo microCT analysis, in which both total lesion volume and mineralized volume were not significantly altered by deletion of *Myd88* in *Pdgfra*-positive cells (Figure 2D–G).

**Figure 3:**
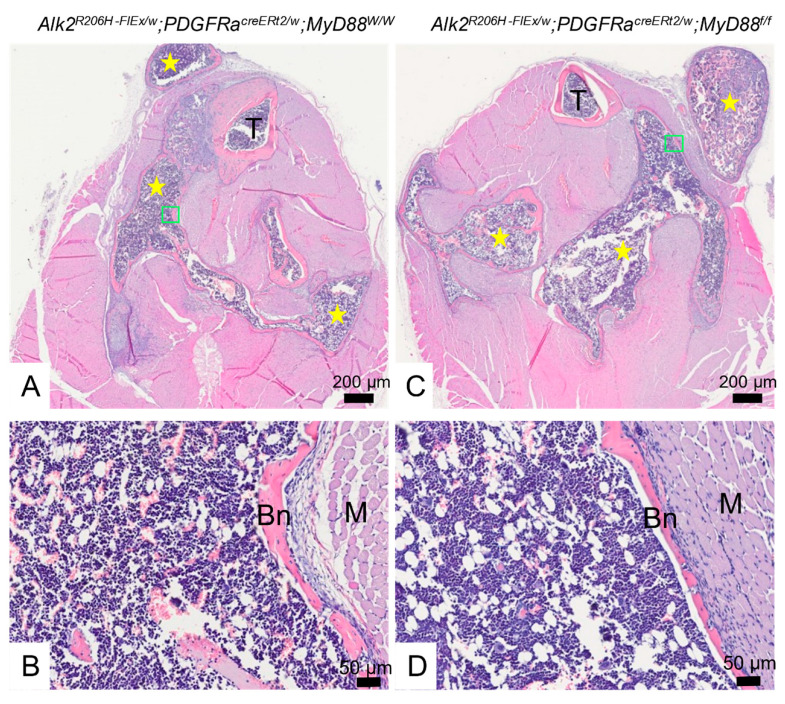
Gross histological examination of EHO in injured hindlimbs of *Acvr1^R206H_FlEx^;MyD88^w/w^* (**A**,**B**) and *Acvr1^R206H_FlEx^;MyD88_Pdgfrα−/−_* (**C**,**D**) mice. The tissue and cellular composition of injured lower hindlimbs was examined in H&E-stained transverse sections of decalcified paraffin embedded specimens collected 21 dpi. Legs from both genotypes consistently contained large amounts of mature intramuscular heterotopic bone (yellow stars in (**A**,**C**)) filled with bone marrow with an unremarkable composition of hematopoietic cells, adipocytes, and erythrocytes (**B**,**D**) that appeared similar to that in host tibia (T). The green boxes in (**A**,**C**) mark the approximate locations of the images in (**B**,**D**). Bn = bone, M = muscle, and T = tibia.

Following the microCT scanning, the maturity and composition of the EHO lesions was examined histologically in H&E-stained sections. In *Acvr1^R206H_FlEx^;Myd88^w/w^* mice, EHO lesions had transitioned from cartilage to ossicles of thin lamellar bone surrounding bone marrow, with few or no trabeculae (Figure 3A,B), as is typical in this model. Careful examination of similar sections from *Acvr1^R206H_FlEx^;Myd88_Pdgfrα-/-_* mice (Figure 3C,D) did not reveal any gross differences in the maturity, structure, or composition of the EHO compared to the *Acvr1^R206H_FlEx^;Myd88^w/w^* mice. While a detailed analysis might still find minor differences in ossicle remodeling or marrow composition, these gross observations demonstrate that deletion of *MyD88* in Pdgfrα-positive cells did not impair maturation of the EHO in this model.

To confirm these results, a similar study was conducted using *Acvr1^R206H_mg/R206H_mg^* mice in which cre-mediated recombination induced homozygous expression of Alk2^R206H^. Recombination of the *Acvr1^R206H_mg^* and *MyD88^fl^* alleles was confirmed by PCR, as in the previous study (Figure S4A,B). As in the *Acvr1^R206H_FlEx^* mice, the area of EHO measured in radiographs at 14 and 21dpi did not significantly differ between groups (Figure S5A–C). Likewise, total lesion volume and mineralized volume of the EHO at 21 days post-injury were not significantly altered by deletion of *MyD88* in cells of the Pdgfrα-positive lineage (Figure S5D–G). Therefore, MyD88-dependent signaling in intramuscular FAPs and other mesenchymal cells does not appear to be critical for EHO in FOP mice.

### 3.3. Global Deletion of MyD88 does Not Alter Muscle Injury-Induced EHO Formation in FOP Mice

MyD88 is best known for its roles in mediating TLR, and IL1R1 inflammatory signals in innate immune cells, including monocytes and macrophages, have important but poorly characterized roles in multiple mouse models of genetically driven and traumatic EHO [24,25,26,49,50,51], including FOP mice [25]. Therefore, muscle injury-induced EHO was studied in *Acvr1^R206H_FlEx^;Myd88^w/w^* and *Acvr1^R206H^^_FlEx^;Myd88^−/−^* mice in which expression of *Alk2^R206H^* and deletion of *MyD88^fl^* were globally induced using R26cre^ERt2^. Recombination of both *Acvr1^R206H_FlEx^* and *Myd88-flox* alleles prior to injury was confirmed in all mice via conventional PCR of gDNA from ear punches (Figure S6A,B). Mice of both genotypes remained healthy and gained weight throughout the 21-day post-injury period, with similar changes in bodyweight between genotypes except for a transient divergence at 14 dpi (Figure S6C).

**Figure 4:**
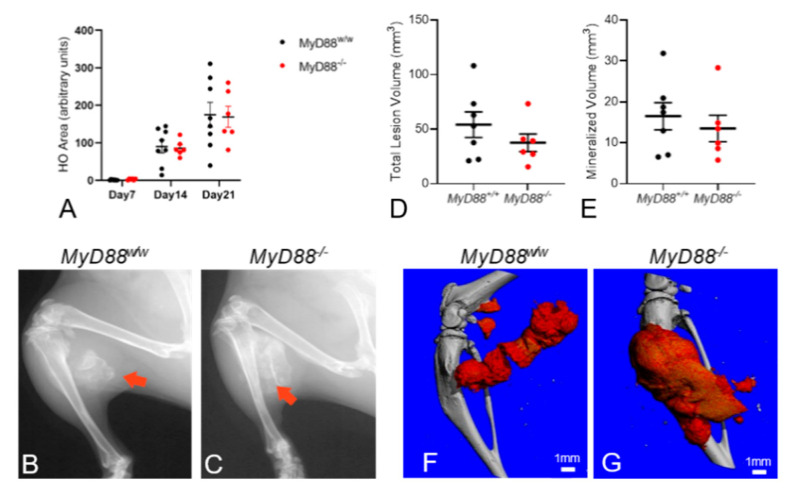
Global deletion of MyD88 in *Acvr1^R206H_FlEx/wt^* FOP mice did not affect muscle injury-induced EHO lesions. (**A**) Semiquantification of intramuscular HO area in in vivo radiographs at 7, 14, and 21 dpi. μCT-based quantification of HO demonstrated that (**D**) total lesion volume and (**E**) mineralized volume were unaffected in *MyD88^−/−^* FOP mice compared to *MyD88^w/w^* FOP mice. Representative radiographs (**B**,**C**) and μCT reconstructions (**F**,**G**) of legs at 21 dpi in *MyD88^w/w^* and *MyD88^−/−^* FOP mice illustrated large intramuscular HO (red arrow and red material). Statistical significance was tested using Kruskal–Wallis test or a two-tailed unpaired Student’s *t*-test. No significant differences were found.

In vivo radiograph analysis revealed formation of extensive HO in both *Acvr1^R206H_FlEx^;Myd88^w/w^* and *Acvr1^R206H_FlEx^;Myd88^−/−^* FOP mice during the 21-day post-injury period, but, surprisingly, the area of radiographic HO was not different between genotypes (Figure 4A–C). This was confirmed by ex vivo microCT analysis, which also did not demonstrate significant differences in lesion volume or mineralized volume (Figure 4D–E). Together, these data suggest that, even in myeloid and other cell types, MyD88 and MyD88-dependent signaling do not have a significant role in the formation of EHO in FOP mice.

**Figure 5:**
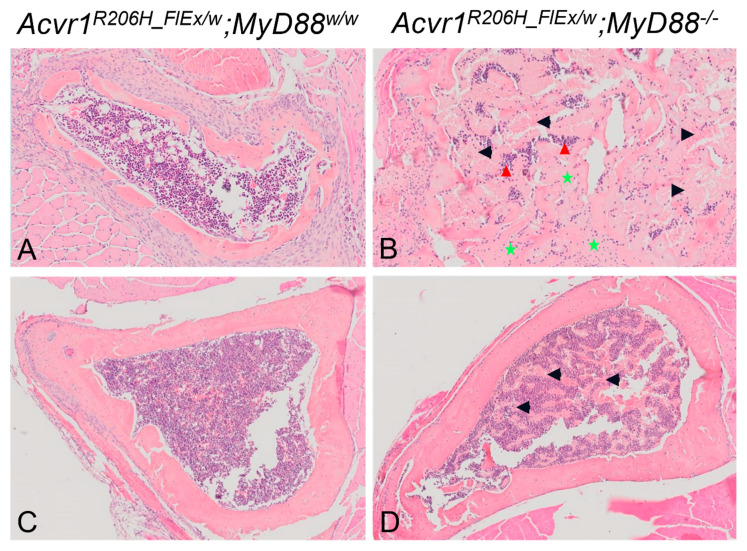
Representative H&E-stained sections of intramuscular EHO lesions and host tibias in *Acvr1^R206H_FlEx^;Myd88^w/w^* and *Acvr1^R206H_FlEx^;Myd88^−/−^* FOP mice 21 dpi. Images are representative micrographs of intramuscular EHO lesions (**A**,**B**) and host tibias (**C**,**D**) in the same sections of decalcified paraffin embedded sections stained with H&E. (**A**) A typical mature ossicle of EHO in the hindlimb muscle of an *Acvr1^R206H_FlEx^;Myd88^w/w^* mouse. While most EHO ossicles in *Acvr1^R206H_FlEx^;Myd88^−/−^* mice were similar to that shown in panel A, some ossicles in *Acvr1^R206H_FlEx^;Myd88^−/−^* mice exhibited a disorganized structure (**B**). Note the striking differences in bone organization and soft tissue composition inside the ossicles, including the presence of dysplastic immature immune cells (red arrow heads), excessive erythropoiesis (black arrows), and areas of extracellular matrix accumulation (green stars). (**C**,**D**)—Lox magnification images of tibiae adjacent to the lesions in (**A**,**B**), illustrating bone marrow composition similar to that of the respective EHO.

While this study was not designed for detailed histomorphometric analysis of bone remodeling within HO, H&E-stained sections of the decalcified hindlimbs were qualitatively assessed for obvious differences in HO morphology, maturation, marrow composition, and resorption of mineralized cartilage and bone. Lesions in *Acvr1^R206H_FlEx^;Myd88^w/w^* mice exhibited the typical EHO morphology and composition seen at 21 days (Figure 5A) as in the previous study above. Mature heterotopic ossicles contained a primarily hematopoietic bone marrow that included adipocytes and scattered areas of erythropoiesis with little or no trabeculated bone structures. In *Acvr1^R206H^^_FlEx^;Myd88^−/−^* mice, HO was also clearly endochondral and most of the HO lesions were composed similar to those in *Acvr1^R206H_FlEx^;Myd88^w/w^* mice. However, some of the ossicles in these mice lacked a well-defined cortex-like shell, and the bone appeared as disorganized and disconnected trabeculae surrounded by dysplastic bone marrow, excessive erythropoiesis, and acellular areas of extracellular matrix (ECM) (Figure 5B). The composition of marrow in EHO ossicles was consistent with the bone marrow phenotypes seen in the respective intact host bones (Figure 5E,F). This suggests the dysplastic marrow associated with EHO lesions of *MyD88^−/−^* mice is not unique to the EHO process but rather a symptom of the known effects of MyD88 deletion on hematopoietic cells [52,53,54,55,56].

Less mature areas of EHO in *MyD88^w/w^* mice also included areas in which mineralized cartilage was rapidly being remodeled to lamellar bone and marrow-filled medullary spaces (Figure 5C). As expected, these areas contained a high density of multinucleated osteoclasts (OCL), the bone surface was predominantly scalloped and eroded, and groups of bone forming osteoblasts (OB) were seen in close proximity. Resorbed space between the remaining bone was rapidly filling with a mixture of immune cells similar to typical healthy bone marrow. In contrast, similar areas of transition from cartilage to bone in *MyD88^−/−^* FOP mice were wider than in *MyD88^w/w^* FOP mice and contained few small osteoclasts, and the resorption spaces were dominated by fibroblasts and osteogenic cells rather than hematopoietic cells (Figure 5D). Resorption areas that were large enough for marrow cell invasion were often filled with acellular matrix, resembling collagen deposition (Figure 5D). While these histological observations suggest the remodeling of cartilage to bone may be impaired in *MyD88^−/−^* FOP mice, this study demonstrates that MyD88-dependent signaling has a surprisingly limited, if any, role during EHO in FOP.

## 4. Discussion

FOP flares and the ensuing formation, remodeling, and expansion of heterotopic ossification involve complex interactions between numerous cell types that are most notably of migratory hematopoietic lineages [25,50,57,58,59] and mesenchymal lineages that derive from both local intramuscular and distant stem cell populations [9,10,13,19,48]. Since the cellular responses to TLR and IL1R1 ligands vary greatly according to cell type, the mechanisms by which their signaling pathways regulate EHO, if any, must be determined in a linage-specific manner.

*Pdgfrα*+ intramuscular FAPs appear to be the primary source of chondrogenic progenitors in FOP, and expression of Alk2^R206H^ in FAPs is required for HO formation in FOP mice [9,10]. Previous reports demonstrated that TLR and IL1R1 signaling, activated by LPS and IL-1β, respectively, enhanced BMP-induced phosphorylation and/or transcriptional activity of Smad1/5/8 in fibroblasts from FOP patients [31] and other cell types [28,29,36,37] but did not utilize intramuscular FAPs. Therefore, the observation that LPS or IL-1β enhanced aberrant ActA-induced pSmad1/5 in FAPs from FOP mice (Figure 1) is novel but not surprising. While FOP flares are a sterile process that does not appear to involve exposure to bacterial LPS, it is widely used as a broad activator of TLR signaling pathways via TLR2 and TLR4, which are also activated by multiple DAMPs [60,61]. The broad consistency among in vitro systems provided a strong rationale for the hypothesis that one or more pathways activated by TLRs and IL1R1 may contribute to chondrogenesis of FAPs in FOP mice.

Although not required for all TLR and IL1R1 signaling, the adapter protein MyD88 was an attractive target for initial in vivo studies interrogating the roles of these pathways in FOP. TLR and IL1R1 ligands released from damaged tissues may be composed of up to three dozen unique molecules [60,61,62], making ligand-targeted studies unreasonable. Even when focusing on LPS and IL-1β, the ligands activate a variety of redundant receptors and intracellular signaling pathways, preventing any one approach from blocking all potential signals. However, MyD88 is a required adapter protein in most, but not all, LPS- and IL-1β-activated signaling pathways, making it the most reasonable first target for in vivo studies aimed at determining a potential role for TLR and/or IL1R1 activation in FOP.

The current studies utilized inducible models of MyD88 deletion in an attempt to separate a potential direct role for MyD88 signaling from indirect effects via the hematopoietic phenotype of germline MyD88 knockout mice. MyD88 plays essential roles in the early stages of hematopoiesis as well as the terminal differentiation of both myeloid and lymphoid cells. Dysfunction of MyD88 has also been linked to multiple hematopoietic disorders. For example, significantly lower antibody responses after vaccination were detected in MyD88-knockout mice [52], whereas enhanced activation of TLR-MyD88-initiated signaling appears to contribute to the pathogenesis of myelodysplastic syndrome [63]. MyD88 is also involved in osteoclast function induced by IL-1β and LPS, and *MyD88*-deficient mice are osteopenic with reduced bone resorption and formation. [64]. Reports that osteoclasts may contribute to EHO in FOP mice [65,66], provide another possibility for indirect effects of long-term *MyD88* deletion. The effects may also provide an explanation for the histological observations in the ossicles of *Acvr1^FlEx+R206H^;MyD88^−/−^* mice.

Thus, the data in Figure 2 and Figure S5 clearly demonstrating that deletion of MyD88 in Pdgfrα-positive FAPs did not alter EHO were rather surprising. Moreover, the failure of global MyD88-deletion to alter injury-induced EHO in FOP mice (Figure 4) was entirely unexpected given the well-established roles of MyD88 in inflammatory cells known to be important in FOP [25,50,57,58].

When interpreting negative results, it is important to carefully examine experimental controls and limitations of the studies. One such limitation is the inability to directly confirm deletion of the *MyD88^fl^* alleles in the FAPs of *Pdgfra^cE2^* mice in which HO was measured. However, Figures S3 and S4 demonstrate a high rate of *MyD88* deletion in intramuscular FAPs in vivo, but not in ear punches, from tamoxifen-treated *MyD88_Pdgfra−/−_* mice. Cre-mediated recombination of the conditional *Alk2^R206H^* alleles in Pdgfrα-positive cells is required for injury-induced EHO in these models. Therefore, robust EHO is also a reliable surrogate for effective cre-mediated recombination in the targeted FAP population. Hence, it is very unlikely that the results of those experiments can be explained by inefficient MyD88 deletion. Finally, global recombination of the *Alk2^R206H_FlEx^* allele and deletion of *MyD88^fl^* alleles in the *R26^cE2/cE2^* mice was confirmed using ear tissue collected at the time of muscle injury (Figure S6). Together, these controls demonstrate a high rate of *MyD88* deletion in the targeted cells in each experiment and support the conclusion that MyD88 and MyD88-dependent signaling are dispensable for muscle injury-induced EHO in FOP mice.

The likely elimination of MyD88-dependent pathways as contributors to FOP flares does not contradict or reduce the potential importance of crosstalk between aberrant BMP signaling and DAMP-induced inflammatory signals. Given the wealth of research in tissue repair and inflammation, it is difficult to envision a logical scenario in which all TLR and IL1R1 signaling is unnecessary for the misdirected muscle repair processes in FOP. Hence, the current studies provide important evidence that substantially reduces the number of candidate signaling pathways. Assuming that LPS signaling is, in fact, transduced only by TLRs, only TRAM and TRIF are known to activate MyD88-independent signaling at this time [67]. Interestingly, TLR4 can activate ECSIT, which was previously reported to mediate TLR/BMPR crosstalk [27,31], via the MyD88-independent TRIF > RIP1 > Ubc13 pathway. IL-1β/IL1R1 can also activate a MyD88-independent pathway involving an IRAK2/TRAF6/ECSIT complex leading to activation of a MAPK cascade [68,69]. Thus, while the hypothesis for the current studies appears to have been disproven, they have narrowed the number of potential TLR and IL-1β signaling pathways that may mediate innate inflammatory crosstalk with BMP signaling and contribute to FOP flares and EHO.

## Data Availability

The data presented in this study are available in the article and Supplementary Material.

## Supplementary Materials

The following are available online at https://www.mdpi.com/article/10.3390/biomedicines9060630/s1, Figure S1: Workflow of semiautomated microCT contouring process, Figure S2: Isolation of FAPs and in vitro recombination of Alk2^R206H_FlEx^ allele, Figure S3: Post-injury changes in body weight and representative tamoxifen-induced recombination in Pdgfra^cE2^;Acvr1^R206H_FlEx/w^;MyD88 mice, Figure S4: Post-injury changes in body weight and representative tamoxifen-induced recombination in Pdgfra^cE2^;Acvr1^R206H_mg/R206H_mg^;MyD88 mice, Figure S5: Conditional deletion of MyD88 in PDGFRα+ cells did not alter injury-induced HO formation in Acvr1^R206H_mg^ homozygous FOP mice, Figure S6: Post-injury changes in body weight and representative tamoxifen-induced recombination in R26^cE2/cE2^; Acvr1^R206H_FLEx/w^; Myd88 mice, Table S1: Primers used for genotyping by conventional PCR, Table S2: Fluorophore compensation cocktails used in FACS isolation of skeletal muscle FAPs, Table S3: Antibodies used for Western blots.
