# Supplementary material for: MyD88 Is Not Required for Muscle Injury-Induced Endochondral Heterotopic Ossification in a Mouse Model of Fibrodysplasia Ossificans Progressiva
Source: Biomedicines. 2021 Jun 1;9(6):630. doi: 10.3390/biomedicines9060630 (PMC8227787; doi:10.3390/biomedicines9060630)
Supplement: Supplementary file 1 [file biomedicines-09-00630-s001.zip › biomedicines-1227254-supplementary.pdf]

biomedicines

MDPI

Supplemental Information

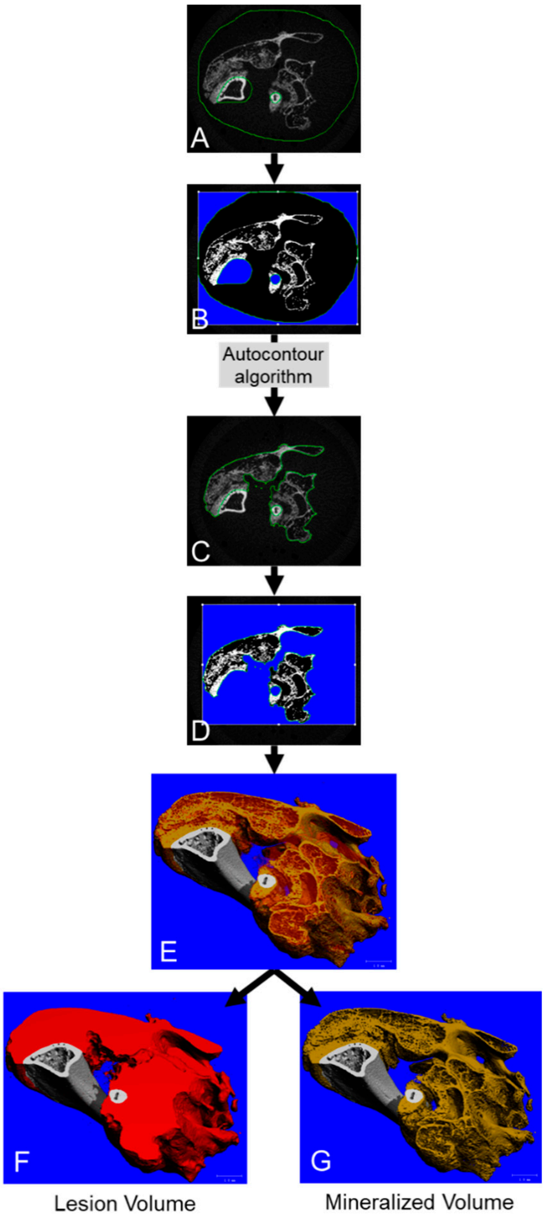

Figure S1. Workflow of semi-automated microCT contouring process. The resulting masks and objects were used to determine lesion volume (LV), mineralized volume (MV), and calculate MV/LV of EHO in microCT scans. Loose contours of the VOI that carefully exclude the host tibia and fibula (A and B) are hand drawn on intermittent 2D slices and a standard "morphing" process is used to interpolate contours in the intervening slices (not depicted). An adaptation of the autocontouring algorithm by Buie et al. [41] is used to fill gaps in the perimeter of the heterotopic bone and create a tight-fitting contour of the outer edge of the mineralized HO (C) that excludes the host tibia and fibula (D and grey in E-F), thus defining the total "lesion volume" (red in F). Standard threshold-based segmentation is used to define mineralized tissue inside the lesion contour and thus define the "mineralized volume" (yellow in G).

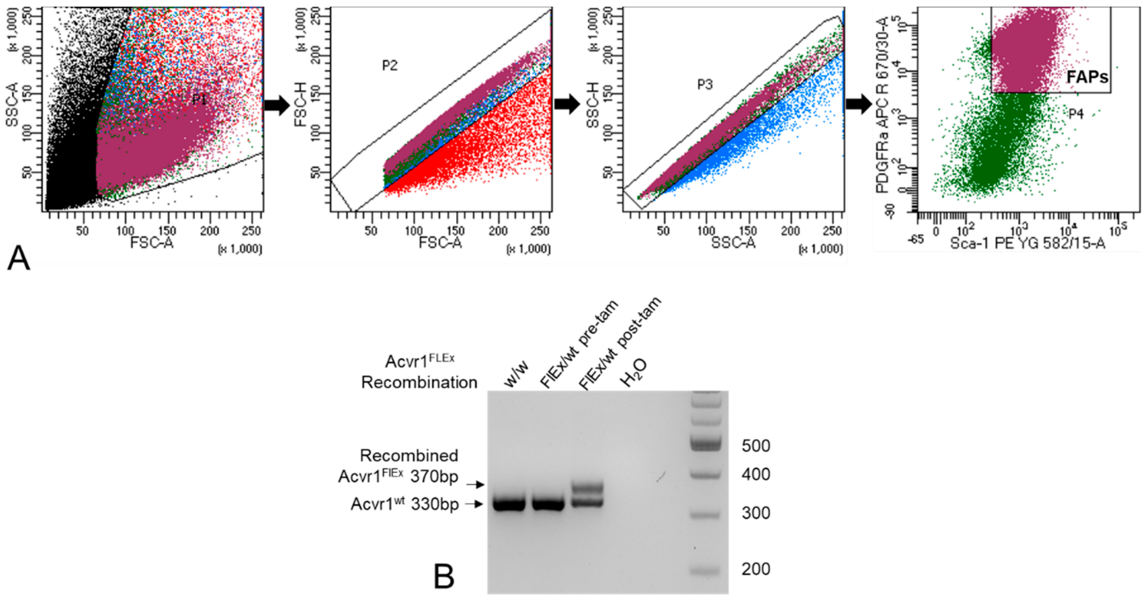

Figure S2. Isolation of FAPs and in vitro recombination of *Acvr1*$^{R206H\_FlEx}$ allele. (A) Scatter plots of the gating strategy used to collect Sca-1+/Pdgfrα+ FAPs from digested muscle tissue following depletion of CD45+, Tert1+, and CD31+ cells by MACS. (B) Conventional PCR products separated by electrophoresis in an agarose gel demonstrating 4OHT-induced recombination of *Acvr1*$^{R206H\_FlEx}$ in the FAPs used in Figure 1.

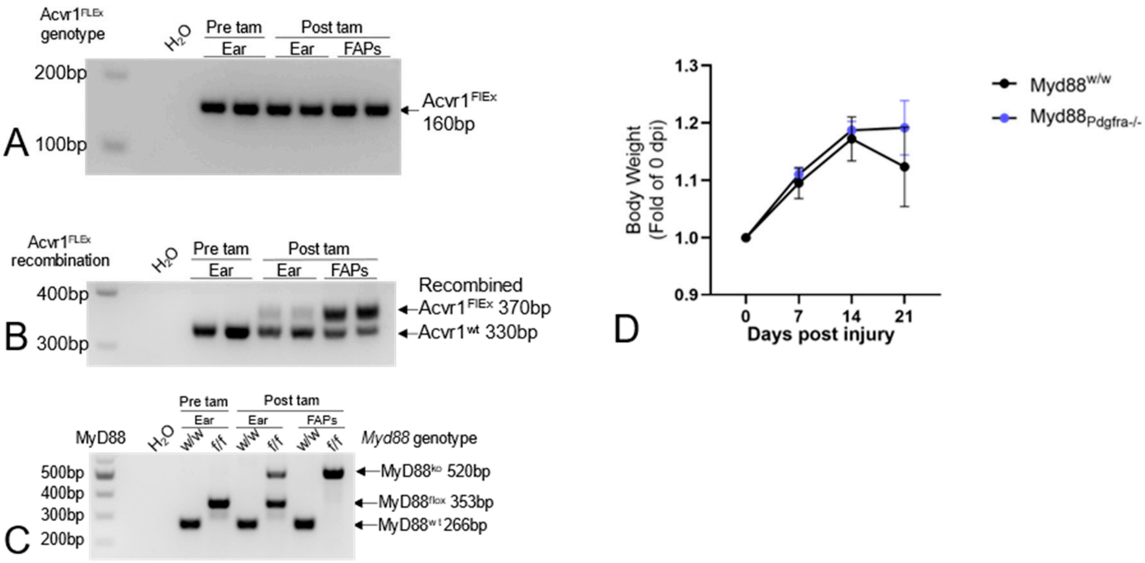

Figure S3. Post injury changes in body weight and representative tamoxifen-induced recombination in *Pdgfra*^{cE2};*Acvr1*$^{R206H\_FlEx/w}$;*MyD88* mice. (A-C) FAPs were harvested from tamoxifen treated *Pdgfra*^{cE2};*Acvr1*$^{R206H\_FlEx/w}$;*MyD88*$^{w/w}$ and *Pdgfa*^{cE2};*Acvr1*$^{R206H\_FlEx/w}$; *MyD88*$^{f/f}$ *mice.* Tamoxifen-induced, cre-mediated recombination of *Acvr1*$^{R206H\_FlEx}$ (A and B) or *MyD88*^{fl} alleles (C) in *Pdgfa-positive* cells was detected by conventional PCR and products separated by electrophoresis in an agarose gel. (D) Change in bodyweight relative to individual weight at the time of muscle injury for all mice in the study shown in Figure 2.

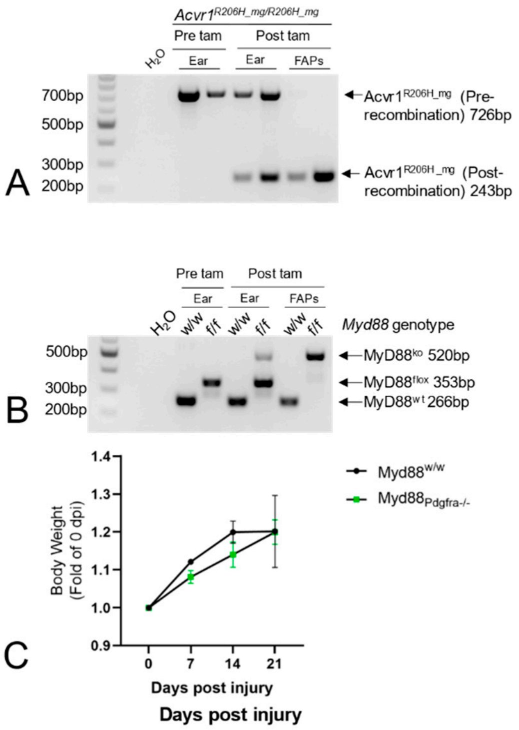

Figure S4. Post injury changes in body weight and representative tamoxifen-induced recombination in *Pdgfra*^{cE2};*Acvr1*$^{R206H\_mg/R206H\_mg}$;*MyD88* mice. (A and B) FAPs were harvested from tamoxifen treated *Pdgfra*^{cE2};*Acvr1*$^{R206H\_mg/R206H\_mg}$; *MyD88*$^{w/w}$ and *Pdgfra*^{cE2};*Acvr1*$^{R206H\_mg/R206H\_mg}$; *MyD88*$^{f/f}$ *mice.* Tamoxifen-induced, cre-mediated recombination of *Acvr1*$^{R206H\_mg}$ (A) and *MyD88*^{fl} alleles (B) in *Pdgfra*+ cells was detected by conventional PCR products separated by electrophoresis in an agarose gel. (C) Change in bodyweight relative to individual weight at the time of muscle injury for all mice in the study shown in Supplemental Figure 5.

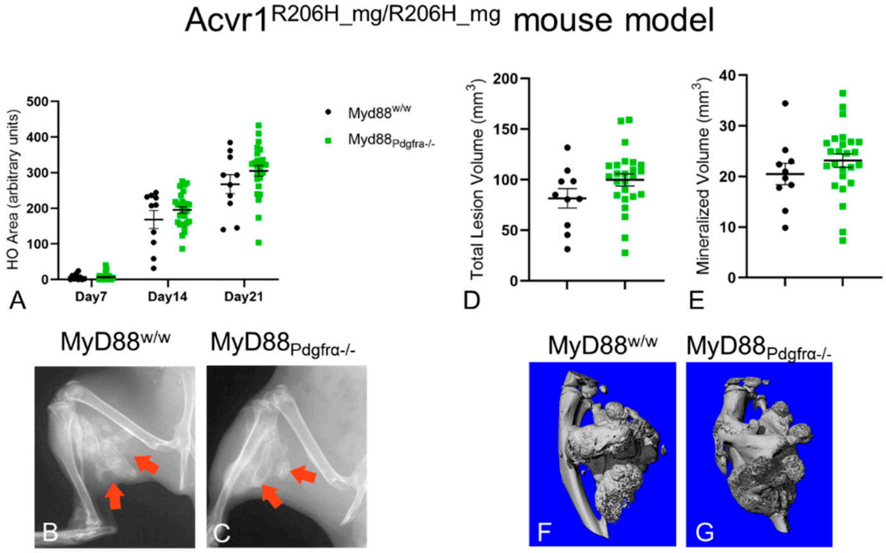

Figure S5. Conditional deletion of MyD88 in PDGFRα-positive cells did not alter injury-induced HO formation in *Acvr1*$^{R206H\_mg}$ homozygous FOP mice. (A) Semi-quantification of intramuscular HO area in radiographs revealed unaffected HO formation in MyD88$_{Pdgfr\alpha-/-}$ FOP mice vs. MyD88$^{w/w}$ FOP mice at 14 and 21 dpi. (D and E) µCT based quantification of HO demonstrated (D) unaffected total lesion volume and (E) mineralized HO volume

in MyD88Pdgfrα-/- FOP mice vs. MyD88$^{w/w}$ FOP mice at 21dpi. Representative radiographs (B and C) and μCT reconstructions (F and G) of legs at 21 dpi illustrating extensive EHO (red arrows) in both genotypes. Statistical significance was tested using Kruskal-Wallis test or a two-tailed unpaired Student's t-test. No significant differences were found.

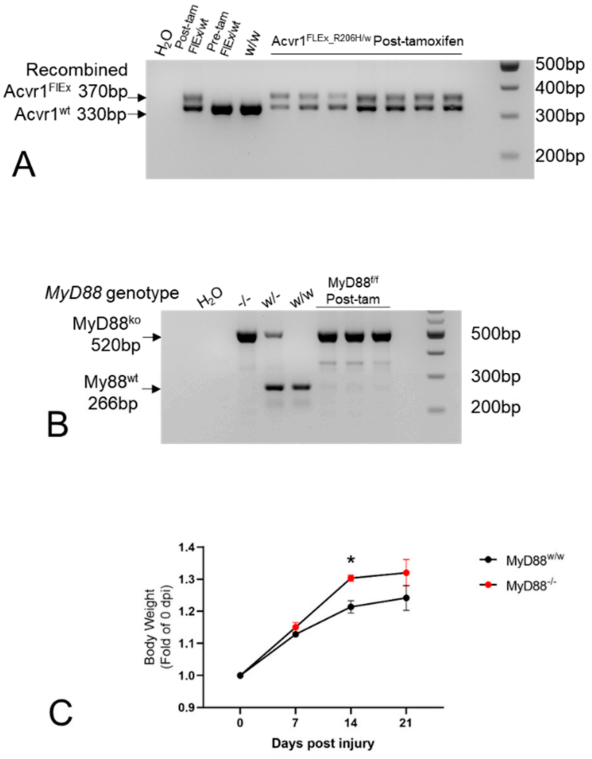

Figure S6. Post injury changes in body weight and representative tamoxifen-induced recombination in *R26$^{cE2/cE2}$;Acvr1$^{R206H_FLEx/w}$;Myd88* mice. Tamoxifen-induced, cre mediated recombination of (A) *Acvr1^{R206H_FlEx}* and (B) *Myd88^{fl}* alleles in representative mice in Figure 4 was detected by conventional PCR products separated by electrophoresis in an agarose gel. (C) Change in bodyweight relative to individual weight at the time of muscle injury for all mice in the study shown in Figure 4. Statistical significance was determined by multiple unpaired t-test with correction for multiple comparisons. (*$p < 0.05$)

Table S1. Primers used for genotyping by conventional PCR

Gene	Primer Name	Sequence(5'→3')
Acvr1^{R206H_FlEx}	COIN4F	AAGATGGAACTTGGAAAATCTGC
	COIN4R	CAGGGCTTGGAAAAGTCTGC
	NOS3A	CTCCAACTTAGTGCAGGTCT
	NOS3B	ATGGTTGCCTTCACACGCTT
Acvr1^{R206H_FlEx} (post recombination)	R206H-Recom-F	TGTATTGCAGGACGCTGAAG
	R206H-Recom-R	CCCCTGAAGTGGAATAACCA
Acvr1^{R206H_mg}	Orris 1848 primer_01F	ATAGTAGACAATGCCCAGCTCTTG
	Orris 1848 primer_02R	GAGAATCACAACCAACATTGCCTG
	Orris 1848 primer_03F	CAGGATGATCTGGACGAAGAGC
	Orris 1848 primer_04R	AAGGAAACAGAGCTGACACGTG
MyD88^{flox}	oIMR9481 WT Forward	GTTGTGTGTGTCCGACCGT
	oIMR9482 Common Reverse	GTCAGAAACAACCACCACCATGC
MyD88 Knock-out	oIMR9481 WT Forward	GTTGTGTGTGTCCGACCGT
	oIMR9482 Common Reverse	GTCAGAAACAACCACCACCATGC
	9335 KO Forward	CCACCCTTGATGACCCCCTA
R26^{cE2}	14314	CGGTTATTCAACTTGCACCA
	oIMR9020	AAGGGAGCTGCAGTGGAGTA
	oIMR9021	CCGAAAATCTGTGGGAAGTC
PDFGRα^{cE2}	PDGFRα WT 43655F	GCCTTAAGCTGGGACATGCT
	PDGFRα 43656R	AGGCCCACAGAACATGGAC
	PDGFRα Mut 16504F	ATCGCATTCCTTGCAAAAGT

Table S2. Fluorophore compensation cocktails used in FACS isolation of skeletal muscle FAPs

PE compensation	APC compensation
100μL of MACS buffer	100μL of MACS buffer
5μL PE anti-PDGFRa Miltenyi REA Ab	3μL APC anti-Sca1 Miltenyi REA Ab
One drop anti-REA beads (MACS Comp Bead Kits, anti-REA, 130104693, Miltenyi Biotec)	One drop anti-REA beads
One drop blank beads (MACS Comp Bead Kits, anti-REA, 130104693, Miltenyi Biotec)	One drop blank beads

Table S3. Antibodies used for Western blots

Target	Host	Dilution factor	Company	Catalog Number
Phospho-Smad1/5 (Ser463/465) (41D10)	Rabbit	1:400	Cell signaling Technology	#9516
Anti-SMAD1+SMAD5 antibody	Mouse	1:500	Abcam	ab75273
Anti-β-Actin antibody	Mouse	1:20000	Sigma Aldrich	A5316
Anti-mouse HRP-linked Antibody	Horse	1:2000	Cell signaling Technology	#7076
Anti-rabbit HRP-linked Antibody	Goat	1:1000	Cell signaling Technology	#7074
